# Endovascular repair of an aortic arch pseudoaneurysm with double chimney stent grafts: a case report

**DOI:** 10.1186/1749-8090-8-80

**Published:** 2013-04-11

**Authors:** Weimin Zhou, Wei Zhou, Jiehua Qiu

**Affiliations:** 1 Department of Vascular Surgery, the second affiliated hospital of Nanchang University, Nanchang, China

**Keywords:** Pseudoaneurysm, Aortic arch, Stent graft, Endovascular repair

## Abstract

Aortic arch pseudoaneurysm is a rare condition but carries a high risk of rupture. We report a case of a 45-year-old man with aortic arch pseudoaneurysm between left common carotid artery (LCCA) and left subclavian artery (LSA), in which a endovascular stent graft combined with double chimneys covered stents were successfully placed. There were no any complaints and complications after 12 months follow-up. The CTA demonstrated thrombus formation in the pseudoaneurysm lumen, no endoleak and the aortic arch, LCCA and LSA were all patent. We feel that the combined endovascular and double chimneys may be a valuable therapeutic alternative when treating aortic arch lesion. However, long-term clinical efficacy and safety have yet to be confirmed.

## Background

Aortic arch pseudoaneurysm is a rare condition but carries a high risk of rupture. Previous reports that we have identified in English literature included conventional surgical repair, hybrid surgery,embolization of an aortic arch pseudoaneurysm with detachable coils and total endovascular debranching of the aortic arch or double-chimney technique [1-7]. Conventional surgical intervention requires a thoracotomy, cardiopulmonary bypass, hypothermic circulatory arrest and aortic cross-clamping, remains a surgical challenge with a high rate of mortality (7-17%) and neurologic complication (4-12%) [1,2,8]. Minimally invasive endovascular repair in treating aortic arch pseudoaneurysm is a better choice. We present a case of aortic arch pseudoaneurysm between left common carotid artery (LCCA) and left subclavian artery (LSA), in which a endovascular stent graft combined with double chimneys covered stents were successfully placed. As we know, there was seldom reported in English literature.

## Case presentation

**Figure 1:**
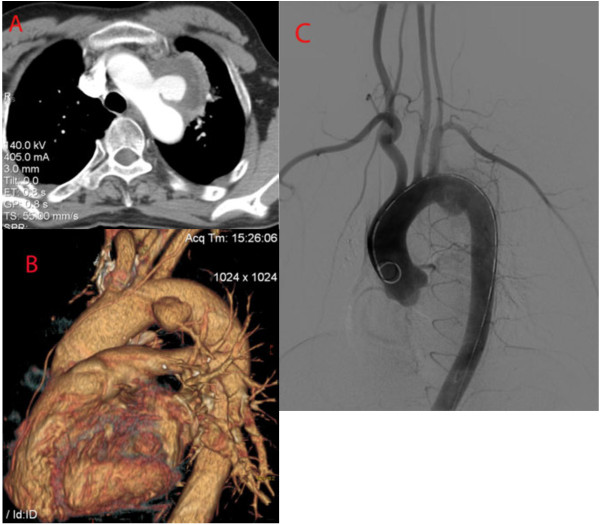
**Preoperative CTA and arteriography: Thoracic CT scan showing an irregular wall at level of aortic arch (A,B).** Preoperative arteriography demonstrated a pseudoaneurysm was evident between the origin of the left common carotid artery and the left subclavian artery; the left vertebral artery was dominant (**C**).

**Figure 2:**
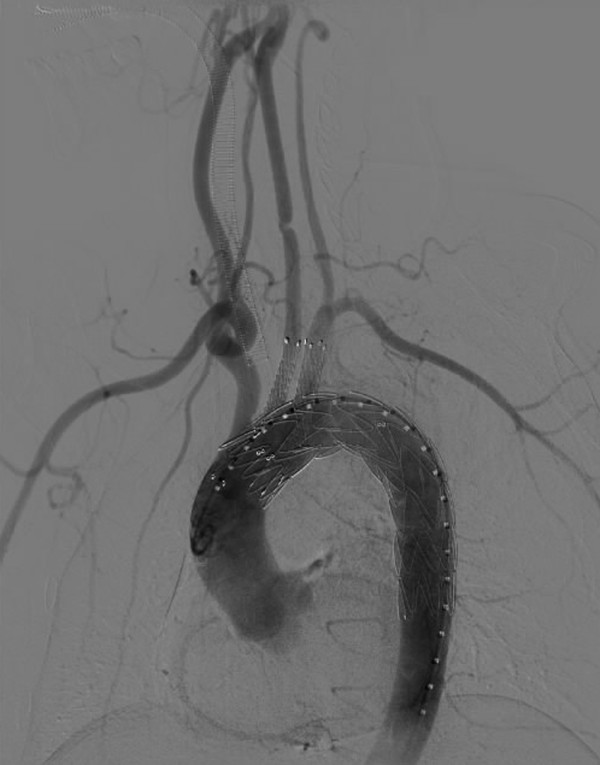
Postoperative arteriography: disappearance of the aortic arch pseudoaneurysm lumen, no evidence of endoleak and patent of the aorta and the branch of aortic arch immediately after deployment.

**Figure 3:**
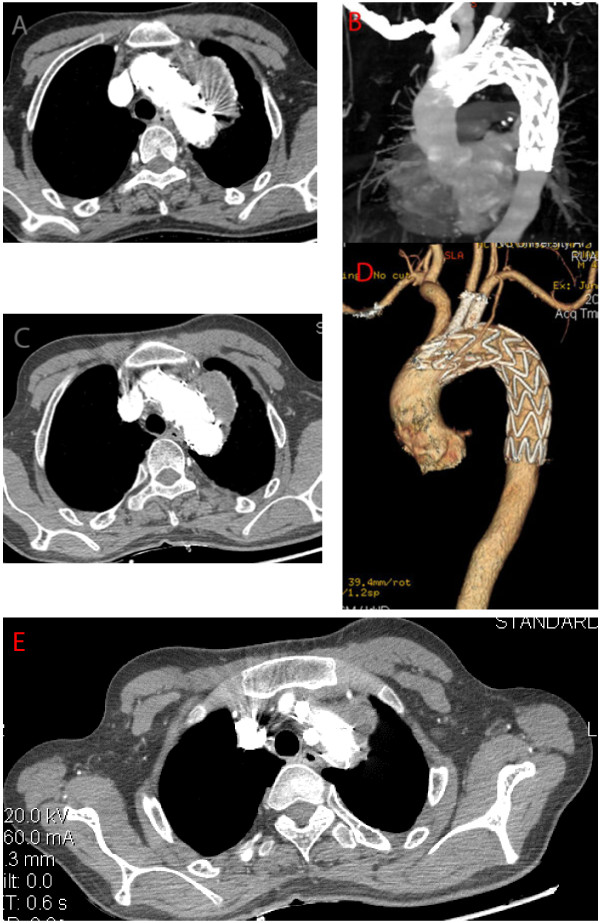
**Follow-up CTA: CTA on the first week post-operative follow-up showed no evidence of proximal endoleak, no filling or enlargement of the pseudoaneurysm (A,B).** Postoperative CTA demonstrated thrombus formation in the pseudoaneurysm lumen, no endoleak and the aortic arch, LCCA and LSA were patent in 12-month follow-up (**C**,**D**,**E**).

A 45-year-old Chinese man was admitted to our center with a chief complaint of hoarseness. He had a history of hoarseness and syncope after a blunt trauma two months prior to presentation. The computed tomographic angiography (CTA) showed an aortic arch pseudoaneurysm between LCCA and LSA (5 mm from LCCA and 0 mm from LSA) with the caliber of tear and the size of pseudoaneurysm lumen being 15 mm and 50 mm, respectively. A digital subtraction angiography (DSA) was performed, and was in agreement with the CTA images. It also demonstrated that the left vertebral artery was dominant (Figure 1). The procedure was performed under general endotracheal anesthesia with full hemodynamic monitoring two days later. The right femoral artery was exposed through a groin oblique incision and cannulated with a 6-French catheter sheath from which an angiographic mark catheter was put into the ascending aortic artery via a 0.035 inch guidewire. The left brachial artery was exposed through a left upper arm longitudinal incision and cannulated with a 8-French catheter sheath from which a pigtail angiographic catheter was inserted into the ascending aortic artery via a 0.035 inch guidewire (Amplatz Super Stiff, Boston Scientific, USA). The LCCA was exposed through a left neck longitudinal incision and cannulated with an 8-French catheter sheath as well. The “C” arm was locked at left anterior oblique (LAO) 45° projection to thoroughly unfold the aortic arch that was cannulated with a 21-French delivery introducer sheath via a 0.035 inch Lunderquist super stiff guidewire (COOK, USA) through the right femoral artery. A thoracic endoprosthesis stent graft (TF2828C150X, VALIANT®, Medtronic Inc., USA)was accurately deployed and the oversize was 5%. Then two covered stents (8 mm × 60 mm, Fluency, FVL08060, BARD, USA) were cannulated with an 8-French delivery introducer sheath via a 0.035 inch Amplatz super stiff guidewire through the left brachial artery and LCCA and was inserted parallel to the stent graft, respectively. The two covered stents were accurately deployed in the proximity of LSA and LCCA to avoid covering the left vertebral artery. Before deployment of the stent grafts, the mean arterial pressure was lowered to 90 mmHg to avoid grafts migration. An aortogram revealed no evidence of endoleak; disappearance of the aortic arch pseudoaneurysm lumen and patent of the aorta and the branch of aortic arch immediately after the stent deployment (Figure 2). The patient recovered from the procedures without complications and was discharged from the hospital in the next three days. Follow-up CTA 1 week later showed no filling or enlargement of the pseudoaneurysm. The endoprosthesis in the aortic arch were patent, without evidence of proximal endoleak. Covered stents in the LCCA and LSA were patent. The patient’s postoperative course was favorable. The hoarseness was significantly alleviated two months later. As of 12 months after endovascular repair, the patient is well without any complaints and complications. Postoperative CTA demonstrated thrombus formation in the pseudoaneurysm lumen, no endoleak and the aortic arch, LCCA and LSA were all patent at 12-month follow-up (Figure 3).

## Discussion

Reported treatment options for aortic arch pseudoaneurysms have included surgical grafts, ligation, pericardial roll graft replacement, embolization with coils, and the use of endovascular stent grafts combined with surgical treatment [1-7,9]. The conventional open surgery was gradually replaced by endovascular treatment due to the complexity of the surgery, surgical trauma and high associated mortality rate. Endovascular treatment is less invasive and is associated with lower morbidity and mortality [3-5]. Since endovascular procedure does not require thoracotomy, circulatory assistance is not necessary and haemorrhages are less likely. What is more, endovascular intervention does not need aortic cross-clamping as such the risk of cerebral, spinal cord and visceral ischemia was decreased. Due to the lower morbidity and mortality rates, thoracic endovascular aortic repair (TEVAR) is considered an acceptable alternative to open surgical repair for patients with various types of aortic diseases. Despite these advantages, TEVAR are technical challenging. The common problem is the presence of an inadequate short proximal and distal landing zone. To achieve an adequate landing zone and sealing zone, the innominate artery, LCCA and LSA need occasionally to be covered. Modification of the stent graft is needed to overcome these limitations of TEVAR. The use of a fenestrated or branched stent graft, which is able to preserve perfusion of the supra-aortic arch vessels, could be one of the alternative approaches [10]. However, a fenestrated or branched stent graft is a custom made device, and is expensive and time consuming to manufacture so they cannot be used in an emergency setting [11,12]. In our patient, coil embolization and endovascular injection of embolic agents were not options because of the caliber of the aorta arch pseudoaneurysm tear (15 mm) and the size of pseudoaneurysm lumen (50 mm). The optimal option for treatment would be TEVAR. However, simple application of TEVAR to treat complicated aortic arch pseudoaneurysm such as in our patient may cause cerebral ischemia and infarction because of the limited landing zone and sealing zone. An alternative approach to this situation is applying the “chimney graft” technique to preserve blood flow to the supra-aortic arch vessels with a short landing zone, that would be impossible to repair with a standard stent graft [13]. The chimney graft is defined as a bare or covered stent that is placed parallel to the main stent graft to preserve blood flow to the supra-aortic arch vessel, which is covered to achieve the proper landing and sealing zone [14]. Since the procedure was introduced, the chimney graft has been successfully applied to preserve the blood flow of the carotid, subclavian, renal and superior mesenteric arteries during endovascular treatment of aortic disease [15-17]. LCCA and LSA covered stents implantation (double chimneys) would be protective of cerebral ischemia or cerebral infarction and subclavian artery steal syndrome. Our successful treatment in this patient suggests that the combined endovascular and double chimneys may be a valuable therapeutic alternative when treating aortic arch lesion, in order to perform a less aggressive surgery and avoid aortic cross-clamping, circulatory assistance and high dose heparinization. Long-term follow-up of a larger number of patients is needed to assess and confirm this favorite result in order to promote this approach. Branched and fenestrated aortic stent graft may be the next approach when it is more convenient, less complicated and available as an off-the-shelf device [14].

## Conclusion

Although early results are promising, long-term clinical efficacy and safety have yet to be confirmed.

## Consent

Written informed consent was obtained from the patient for publication of this Case report and any accompanying images. A copy of the written consent is available for review by the Editor-in-Chief of this journal.

## Competing interests

The authors declare that they have no competing interests.

## Acknowledgements

The authors thank Tom kuang, MD for retouching the manuscript.

## Authors’ contributions

WmZ analyzed and interpreted the patient data. WZ and JQ were the major participants of the operation. All authors read and approved the final manuscript.
